# Using low volume eDNA methods to sample pelagic marine animal assemblages

**DOI:** 10.1371/journal.pone.0303263

**Published:** 2024-05-15

**Authors:** Michelle E. Dan, Elan J. Portner, Jeff S. Bowman, Brice X. Semmens, Sarah M. Owens, Stephanie M. Greenwald, C. Anela Choy

**Affiliations:** 1 Integrative Oceanography Division, Scripps Institution of Oceanography, University of California San Diego, La Jolla, California, United States of America; 2 Marine Biology Research Division, Scripps Institution of Oceanography, University of California San Diego, La Jolla, California, United States of America; 3 Biosciences Division, Argonne National Laboratory, Lemont, Illinois, United States of America; Stockholm University, SWEDEN

## Abstract

Environmental DNA (eDNA) is an increasingly useful method for detecting pelagic animals in the ocean but typically requires large water volumes to sample diverse assemblages. Ship-based pelagic sampling programs that could implement eDNA methods generally have restrictive water budgets. Studies that quantify how eDNA methods perform on low water volumes in the ocean are limited, especially in deep-sea habitats with low animal biomass and poorly described species assemblages. Using 12S rRNA and COI gene primers, we quantified assemblages comprised of micronekton, coastal forage fishes, and zooplankton from low volume eDNA seawater samples (n = 436, 380–1800 mL) collected at depths of 0–2200 m in the southern California Current. We compared diversity in eDNA samples to concurrently collected pelagic trawl samples (n = 27), detecting a higher diversity of vertebrate and invertebrate groups in the eDNA samples. Differences in assemblage composition could be explained by variability in size-selectivity among methods and DNA primer suitability across taxonomic groups. The number of reads and amplicon sequences variants (ASVs) did not vary substantially among shallow (<200 m) and deep samples (>600 m), but the proportion of invertebrate ASVs that could be assigned a species-level identification decreased with sampling depth. Using hierarchical clustering, we resolved horizontal and vertical variability in marine animal assemblages from samples characterized by a relatively low diversity of ecologically important species. Low volume eDNA samples will quantify greater taxonomic diversity as reference libraries, especially for deep-dwelling invertebrate species, continue to expand.

## Introduction

In pelagic ecosystems, community composition directly influences food web structure, biogeochemical cycling, and fisheries landings [1–3]. Monitoring marine animal communities over horizontal and vertical space thus aids in understanding variability in pelagic ecosystem structure and services. However, quantifying pelagic community composition often requires costly ship-based research operations and specialized oceanographic sampling gear [4,5]. Zooplankton and micronekton, comprising most of the animal biomass in pelagic systems [2,3], are commonly sampled with nets and quantifying their diversity requires life stage-specific taxonomic expertise across numerous phyla. Environmental DNA (eDNA) methods have immense potential to expand pelagic animal community sampling by reducing these operational constraints [6,7].

eDNA methods aim to sample the genetic material shed from organisms into their environment (e.g., skin, feces), which can be sequenced and matched to genetic reference libraries to simultaneously detect a range of taxa [6,8,9]. Collecting seawater samples instead of individual animals reduces sampling requirements and sequence-based methods can balance some of the shortcomings of using nets, which are size selective and poor samplers of highly mobile and soft-bodied gelatinous taxa [6,10,11]. Taxonomic assignment of genetic material relies on centralized reference libraries rather than dedicated morphological taxonomic expertise [12,13], and sequencing eDNA at multiple marker genes enables detection of animals spanning a large range of body sizes and types, as well as multiple life history stages [14,15]. When compared to biodiversity estimates using nets and visual surveys, eDNA methods generally detect higher diversity per unit effort but each represents distinct component assemblages of the overall community [16–19]. Although there appears to be agreement between read abundance and relative abundance within animal groups [13,20,21], variability in DNA shedding rates and primer biases among animal groups likely exacerbates variability in community composition estimates among methods [22,23]. As reference libraries expand across multiple markers [12,24,25], eDNA methods will better detect pelagic zooplankton and micronekton diversity and distributions [7,26,27].

Few studies have used eDNA to assess diversity in waters deeper than 200 m and clear methodological challenges are emerging (e.g., [28–30]). Animal diversity in the deep pelagic is poorly sampled relative to epipelagic diversity [15,31] and deep-pelagic taxa are likely underrepresented in reference libraries relative to their shallow-water counterparts. Biomass in deep pelagic waters is generally low and eDNA densities are lower relative to shallow habitats [28,32]. While eDNA collection across depths can be maximized by filtering large volumes of seawater (2–60 L per sample, [28,33]), most pelagic sampling programs allocate water collections to a range of other routine chemical and biological measurements. Pelagic sampling programs that do not explicitly include eDNA collections within restrictive water budgets may have limited volumes remaining for eDNA analyses. For eDNA methods to be broadly useful for monitoring pelagic animal diversity and distributions, we need to examine how eDNA methods resolve diversity across taxonomic groups and habitat depths with a range of water volumes.

Our primary objective was to assess how eDNA samples collected from remaining unallocated water volumes reflect animal assemblage composition in the southern California Current Ecosystem (CCE). We used low volume water samples from the California Cooperative Oceanic Fisheries Investigations (CalCOFI) program–a long-running pelagic ecosystem monitoring program [34]–and collected water samples on an independent survey utilizing depth-discrete net samples in the deep pelagic. We investigate: *i*) the differences between assemblages detected by low volume eDNA and net-based morphological methods, *ii*) the ability of low volume eDNA methods to identify diverse taxonomic groups across shallow and deep habitats and *iii*) whether opportunistic, low volume eDNA samples can describe animal assemblage biogeography in the CCE. We also discuss how low volume eDNA samples could be optimized to provide a broader picture of pelagic animal diversity.

## Materials and methods

### Field sampling

**Fig 1:**
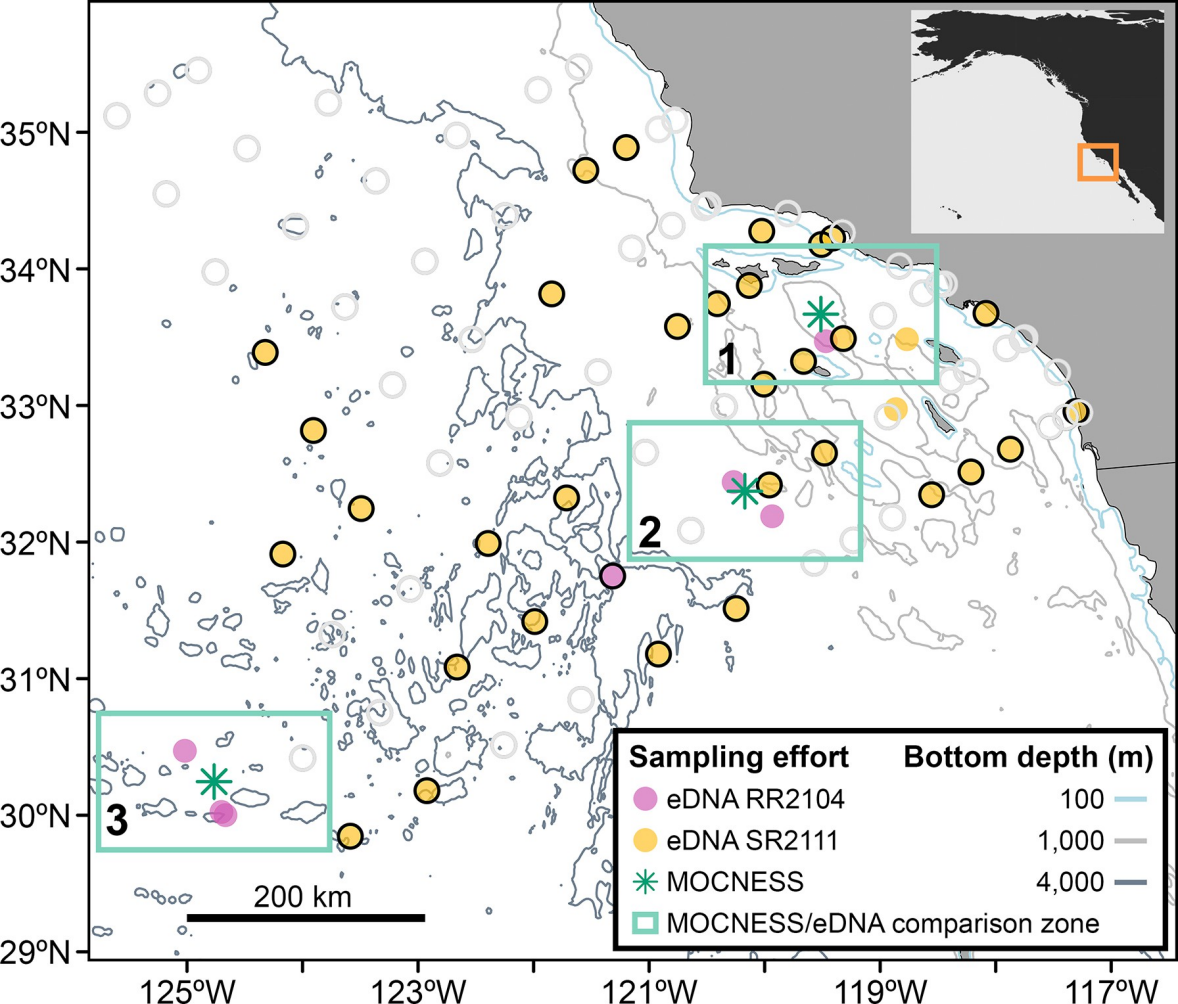
Sampling locations for eDNA collection and MOCNESS trawls. eDNA sampling (closed circles) and MOCNESS trawls (green asterisks) were performed within the Southern California Current Ecosystem, mostly at designated CalCOFI stations (open circles). MOCNESS trawls were conducted at three stations representing distinct pelagic habitats. Three zones (numbered within each green square) are indicated where eDNA samples were collected in proximity to MOCNESS samples. Within these three zones, we compared animal assemblage diversity between sampling methods. Map includes bathymetry data from NOAA National Centers for Environmental Information [35] and coastline data from Natural Earth; all data are in the public domain.

Sampling was performed within the southern California Current ecosystem on two independent research cruises. eDNA samples were collected on the Fall 2021 CalCOFI survey aboard R/V *Sally Ride* (SR2111: October 31-November 14, 2021; 33 total stations, Fig 1 and Table 1). eDNA collection (seven stations) and Multiple Opening/Closing Net and Environmental Sensing System tows (MOCNESS, three stations) were conducted on an independent survey aboard the R/V *Roger Revelle* (RR2104: June 15–27, 2021). No field permits were required for the collections in this study.

**Table 1:** Sampling location, depth, and volume for eDNA samples and MOCNESS trawls.

Method: Zone	Latitude (°N) range (median)	Longitude (°W) range (median)	Distance from coast (km) range (median)	Environmental samples (controls)	Depth (m) range (median)	Volume filtered (mL) range (median)
*eDNA*: *total*	29.8–34.9 (32.6)	-125.0 –-117.3 (-120.2)	109.5–753.5 (276.7)	436 (11)	1.0–2200.0 (100.4)	360–1800 (1000)
*eDNA*: *Zone 1*	32.7–34.3 (33.6)	-120.8 –-117.9 (-119.7)	109.5–240.0 (182.9)	121 (1)	1.0–1500.0 (70.0)	600–1800 (990)
*eDNA*: *Zone 2*	31.4–33.0 (32.4)	-122.0 –-118.2 (-119.5)	233.1–501.2 (282.9)	137 (2)	1.8–1600.0 (120.9)	360–1800 (990)
*eDNA*: *Zone 3*	29.8–30.5 (30.0)	-125.0 –-122.9 (-124.7)	662.2–753.5 (723.0)	39 (2)	2.0–2200.0 (175.0)	650–1800 (1560)
*MOCNESS*: *Zone 1*	33.6–33.8 (33.7)	-119.7 –-119.4 (-119.5)	156.2–180.2 (168.3)	16	4.2–1749.1 (382.4–696.4)	-
*MOCNESS*: *Zone 2*	32.2–32.6 (32.3)	-120.3 –-119.9 (-120.2)	308.8–339.3 (326.2)	6	0.9–1001.6 (199.7–398.4)	-
*MOCNESS*: *Zone 3*	30.1–30.5 (30.3)	-125 –-124.6 (-124.8)	715.9–755.8 (729.5)	5	12.5–718.6 (26–497.1)	-

Zones correspond to the numbered boxes in Fig 1. MOCNESS data reflect all nets that are used in comparison with co-located eDNA samples.

Seawater was collected at each station using 10 L Niskin bottles affixed to a rosette sampler equipped with a conductivity, temperature, and depth (CTD) sensor package (SeaBird Electronics). Following allocation to other analyses, seawater volumes ranging from 360–1800 mL were transferred into sterilized four-liter carboy containers for eDNA analyses from up to 24 different depths per CTD cast (1–2200 m). Carboys were stored at 4°C in the dark until the seawater was filtered for eDNA analyses. Between uses, carboys were rinsed three times with purified MilliQ water, filled with 400–800 mL of 10% hydrochloric acid, and soaked for at least one hour. To distribute acid over the entire inner surface, carboys were shaken vigorously before and after soaking. Finally, the carboys were rinsed three more times with MilliQ water. Seawater was filtered through a 0.2-μm Supor membrane disc filter (47-mm diameter, Pall Corporation, Port Washington, NY, USA) supported on a 500 mL steel funnel attached to a WaterVac vacuum pump (Sterlitech, Auburn, Washington, USA). The filtration setup was cleaned between samples by rinsing with ~50 mL of 95% ethanol followed by ~100 mL of MilliQ water, and the funnel was cleaned twice following the same protocol between CTD casts. Forceps were thoroughly rinsed with 95% ethanol then MilliQ water between eDNA filter samples. MilliQ water was filtered alongside each CTD cast on RR2104 and on four CTD casts on SR2111, serving as eDNA-free negative controls. Filters were placed in sterile 15-mL tubes and stored at -80°C until DNA extraction.

MOCNESS trawls (10 m^2^ mouth area with five depth-discrete nets) were used to sample zooplankton and micronekton from up to five depth layers per trawl (0–1750 m deep) as described in Choy et al. [36]. Briefly, net-collected organisms were identified to the most-specific taxonomic level possible based on morphological characteristics described in published keys. Organisms were then enumerated for each taxonomic group (full dataset to be published elsewhere). The sampling of marine species with MOCNESS trawls was reviewed and approved by the University of California San Diego’s Institutional Animal Care and Use Committee (AAALAC Accreditation #000503) under protocol S21126 to C.A. Choy.

### eDNA extraction, amplification, and sequencing

eDNA filters were sectioned into quarters prior to extraction to maintain source material for future work. DNA was extracted from one quarter of a filter per sample using a KingFisherTM Flex Purification System and MagMAX Microbiome Ultra Nucleic Acid Isolation Kit (ThermoFisher Scientific, Waltham, Massachusetts, United States). To maximize broad detection of animals while minimizing the number of primers applied, we selected mitochondrial 12S ribosomal RNA (12S) and cytochrome oxidase subunit I (COI) genes as barcode regions. Both barcode regions have large reference libraries and effectively identify a broad diversity of invertebrates and vertebrates, respectively [23,32,37–39]. We applied 12S-V5 primers amplifying a 73–110 bp fragment of the 12S gene [40] and the mlCOIintF and jgHCO2198 primers amplifying a 313 bp fragment of the animal COI gene [41]. Primer sequences, PCR reaction recipes, and PCR cycle conditions are given in the Supplementary Information (Methods). Amplicon libraries were sequenced via paired end sequencing (2 X 150bp for 12S and 2 X 300bp for COI) on Illumina’s MiSeq platform at The Environmental Sample Preparation and Sequencing Facility at Argonne National Laboratory.

### Bioinformatics pipeline

Reads were filtered, denoised, and merged in the *dada2* pipeline version 1.26.0 [42]. Default parameters were used except with the ‘filterAndTrim’ command, where trimLeft was set to 15 and truncLen set to 136 for 12S and 225 for COI. Samples represented by fewer than 1,000 reads after being run through the *dada2* pipeline were considered to have been unsuccessfully sequenced and removed from the 12S and COI datasets prior to analyses. The resulting unique amplicon sequence variants (ASVs) were classified in *dada2* against publicly available sequence reference libraries for 12S (*MIDORI2* [43]) and COI (*MetaZooGene* [12]). ASVs matching to taxa that do not occur in marine habitats likely represent contamination or misclassification and were removed from our datasets prior to analyses. ASVs matched to Primates (i.e., human sequences), Galliformes, Rodentia, Bovidae, Suidae, Chiroptera, and Cypriniformes were removed from the 12S dataset. COI reads matching to the bivalves *Bathymodiolus japonicus*, *Saccostrea malabonensis*, and *Abyssogena phaseoliformis* were observed in relatively high numbers in most samples and controls. These taxa, not known from our study, were considered contamination and removed from the COI dataset. All vertebrate sequences, which accounted for less than 3% of decontaminated ASVs, were also removed from COI the dataset because 12S primers are more effective at identifying vertebrate eDNA [32]. To minimize the potential impacts of cross-contamination on our results, all ASVs identified in the negative controls were removed from the environmental samples from each cruise prior to analyses following Govindarajan et al. [44].

### Standardizing eDNA and MOCNESS data

To assess variability in the assemblages detected by eDNA and MOCNESS methods, we standardized the spatial and taxonomic resolutions across methods. We accounted for horizontal and vertical variability in sampling effort between methods by grouping eDNA samples collected within the vicinity of the MOCNESS tows and binning eDNA samples by collection depth to match MOCNESS sampling depths. eDNA samples collected within one degree latitude and two degrees longitude of each MOCNESS sampling site were assigned to the same sampling “zone” (Fig 1). Within each zone, eDNA and MOCNESS samples were assigned to shallow (0–200 m) or deep (200–1750 m) depth bins.

We standardized taxonomic identifications and classifications using the most-specific taxonomic resolution shared between methods. Vertebrate taxonomic identifications were grouped at the order level, except for orders *incertae sedis* within Ovalentaria and Eupercaria which were grouped at the series level [45]. The taxonomic resolution of invertebrate identifications was more variable and invertebrate taxa were binned into broad, mutually exclusive animal groups including phyla and suborders (Fig 3B). Hereafter the term “taxonomic group” is used to refer to these mutually exclusive animal classifications in the eDNA and MOCNESS datasets.

Animal communities are generally quantified using the abundances or biomass of each taxon (e.g., [4]). However, the methods we used do not share a comparable metric of abundance due to numerous issues with connecting ASV read counts to animal abundances across diverse taxa (e.g., variable gene copy numbers [46], differences in eDNA shedding rates [22], primer biases [23,37], and PCR biases [47]). Thus, we quantified presence-based community composition as the “proportional occurrence” of each taxonomic group per sample. For the MOCNESS data, proportional occurrence was quantified as the number of taxa present within each taxonomic group divided by the total number of taxa per sample. For the eDNA data, proportional occurrence was calculated as the number of ASV presences for each taxonomic group divided by the total number of ASVs per sample. This metric reflects variability in the proportional taxonomic richness of each taxonomic group among sampling methods.

### eDNA and MOCNESS data comparisons

To quantify the correlation between assemblages sampled by eDNA and MOCNESS we performed a Mantel test on Morisita-Horn dissimilarities [48] of proportional occurrences among samples using *vegan* (version 2.6–4 [49]) in R. Each unique combination of method, zone, and depth bin contained samples collected during both day and night, but we did not have enough MOCNESS samples to examine temporal variability in the correlation among methods. We present average proportional occurrences (± SD) for each taxonomic group across all zones and depths to provide a standardized summary of the richness sampled by each method.

### eDNA sample variability across the deep pelagic

To understand how the characteristics of eDNA samples vary with depth, we quantified the sample volume-corrected DNA concentration in each sample (measured DNA concentration * (1000 mL/seawater sample volume)). For samples with more than 1,000 reads, we quantified read counts and total ASVs per mL for each primer in each sample. We also calculated the proportion of ASVs assigned to species per sample to determine whether the libraries we used provided variable match probabilities across depths. eDNA sampling density was disproportionately high at depths shallower than 200 m (67% of samples), and samples were binned into three depth groups (0–200 m, 200–600 m, >600 m) to simplify comparisons across our skewed sampling depths. These depth groups reflect ecological variability in our study area, where 200 m and 600 m approximate boundaries of the daytime and nighttime deep scattering layers [50]. When data were non-normal but homoscedastic among depth groups, the effect of depth group on each eDNA metric was examined using a Kruskal-Wallis test. When data were non-normal and heteroscedastic, a Welch’s ANOVA on ranks was used [51,52]. For significant Welch’s ANOVA tests, pairwise comparisons among groups were made using Games-Howell tests on ranks [53].

### Linking ASV diversity to species diversity

In the absence of species-level ASV identifications, many metabarcoding studies examine assemblage composition at the ASV level [19,21,54], but it is not clear how communities of ASVs reflect taxonomic assemblages. If species are commonly represented by multiple ASVs, ASV-level richness would overestimate taxonomic richness and could distort interpretations of spatiotemporal variability in assemblage composition. We examined the relationship between ASV and species diversity by quantifying the number of ASVs assigned to each species per taxonomic group for both primers. The mean number (± SD) of ASVs assigned to each species is reported for each taxonomic group.

### eDNA-based assemblage biogeography

To examine how taxa sampled with eDNA are associated across horizontal and vertical space, we performed hierarchical clustering analysis on the 12S and COI datasets separately. For both primers, only ASVs identified to the species level were included in the clustering analyses to ensure all taxonomic assignments were mutually exclusive. To minimize the influence of poorly sampled species on informative clustering, species present in fewer than three samples were excluded from both the 12S and COI datasets [55]. Mammal and bird ASVs were removed from the 12S dataset to focus on assemblages comprised of zooplankton, micronekton, and coastal forage fishes [56]. To minimize the potential impacts of species that undertake diel vertical migration on our ability to distinguish variability in community compositions across depths [19,29], we limited cluster analyses to eDNA samples collected at night, defined as the period from 90 minutes after sunset to 90 minutes before sunrise.

Presences/absences were assigned to each species and clustering was performed on Jaccard dissimilarities among samples using Ward’s minimum variance criterion [57] in the *stats* package (version 4.2.2 [58]) in R. Up to 10 of the most frequently occurring species per cluster were used to visualize the core community composition in each cluster. To assess the separation distance, or cohesiveness, between resulting clusters, we performed silhouette analysis and quantified the total within-group sum of squares using the *factoextra* package (version 1.0.7 [59]). We used the elbow method on both metrics of separation distance to select an “optimal” number of clusters that provided a parsimonious but informative level of sample partitioning.

Species diversity in each cluster was quantified with species accumulation curves using a random accumulator method in *vegan* (version 2.6–4 [49]) in R. To better understand taxonomic diversity within clusters, we also quantified the average number of species per sample and frequency of occurrence of each species per cluster. Clusters with average silhouette widths ≥0.1 and containing fewer than 15% of samples with negative silhouette widths were considered adequately cohesive and retained in the analysis [60]. We visualized the core geographic distributions of the species assemblages represented in each cluster by mapping the 50% probability density contours of the samples within “shallow” (0–120 m) and “deep” depth bins (121–565 m for 12S; 0–120 m and 121–900 m for COI). Depth bins were selected to depict similar numbers of samples in shallow and deep bins (S1 Fig in S1 File).

## Results

### Overview of animal biodiversity detected by eDNA and MOCNESS methods

A total of 436 eDNA samples from 40 CTD casts and 11 negative controls were extracted and sequenced using 12S and COI primers (Table 1). DNA concentrations averaged 2.76 ng μl^−1^ (SD = 3.05 ng μl^−1^) for environmental samples and were below detection for all negative controls except two at 0.08 ng μl^−1^ (S1 Dataset). Three environmental samples had DNA concentrations below detection levels and were excluded from the analyses. Following the denoising and filtering protocols described in the methods, 231 12S samples (54%) and 331 COI samples (79%) had more than 1,000 reads and were included in our analyses. The mean reads per sample was 24,333 in our 12S dataset and 31,611 in our COI dataset (Table 2). Restricting reads to only marine taxa relevant to the study area resulted in 245 12S ASVs comprising 868,818 reads and 7,971 COI ASVs comprising 616,621 (Table 2). The negative controls contained 235,606 reads of 36 12S ASVs and 128,836 reads of 3,757 COI ASVs (Table 2). However, all ASVs that accounted for >1% of the total read abundance in the negative controls could not be assigned any taxonomy by our bioinformatics pipeline and most of the ASVs (80% of 12S, 91% of COI) and reads (99% of 12S, 86% of COI) present in the negative controls were from non-target taxa or could not be assigned any taxonomy. Seven 12S ASVs and 299 COI ASVs from target marine taxa were present in the negative controls (Table 2). These ASVs represented 732,559 and 78,885 reads in the 12S and COI environmental samples, respectively, and were removed prior to analyses.

**Table 2:** Summary of sampling characteristics for eDNA sequencing and MOCNESS trawls.

	**12S**		**COI**	
	eDNA	Control	eDNA	Control
*Number of samples*	428	11	419	8
*Total reads after dada2 analysis*	7,887,854	235,606	11,039,083	128,836
*Average number of reads per sample* *(± SD)*	24,333 ± 17,443	21,419 ± 24,824	31,611 ± 20,814	21,441 ± 15,566
*Total ASVs*	1,322	36	174,156	3,757
*Total target marine animal reads*	868,818	1,830	616,621	18,085*
*Average number of target marine animal reads per sample (± SD)*	3,761 ± 4,709	166 ± 454	1,964 ± 2,966	3,014 ± 6,914*
*ASVs belonging to target marine animals*	245	7	7,971	299
**MOCNESS**				
	Vertebrates	Invertebrates		
*Number of samples*	27	27		
*Total unique taxa*	68	153		

* A single negative control contained 17,121 reads (95% of the COI negative control reads). The mean number of reads in the remaining seven COI negative controls is 193 ± 247.

**Fig 2:**
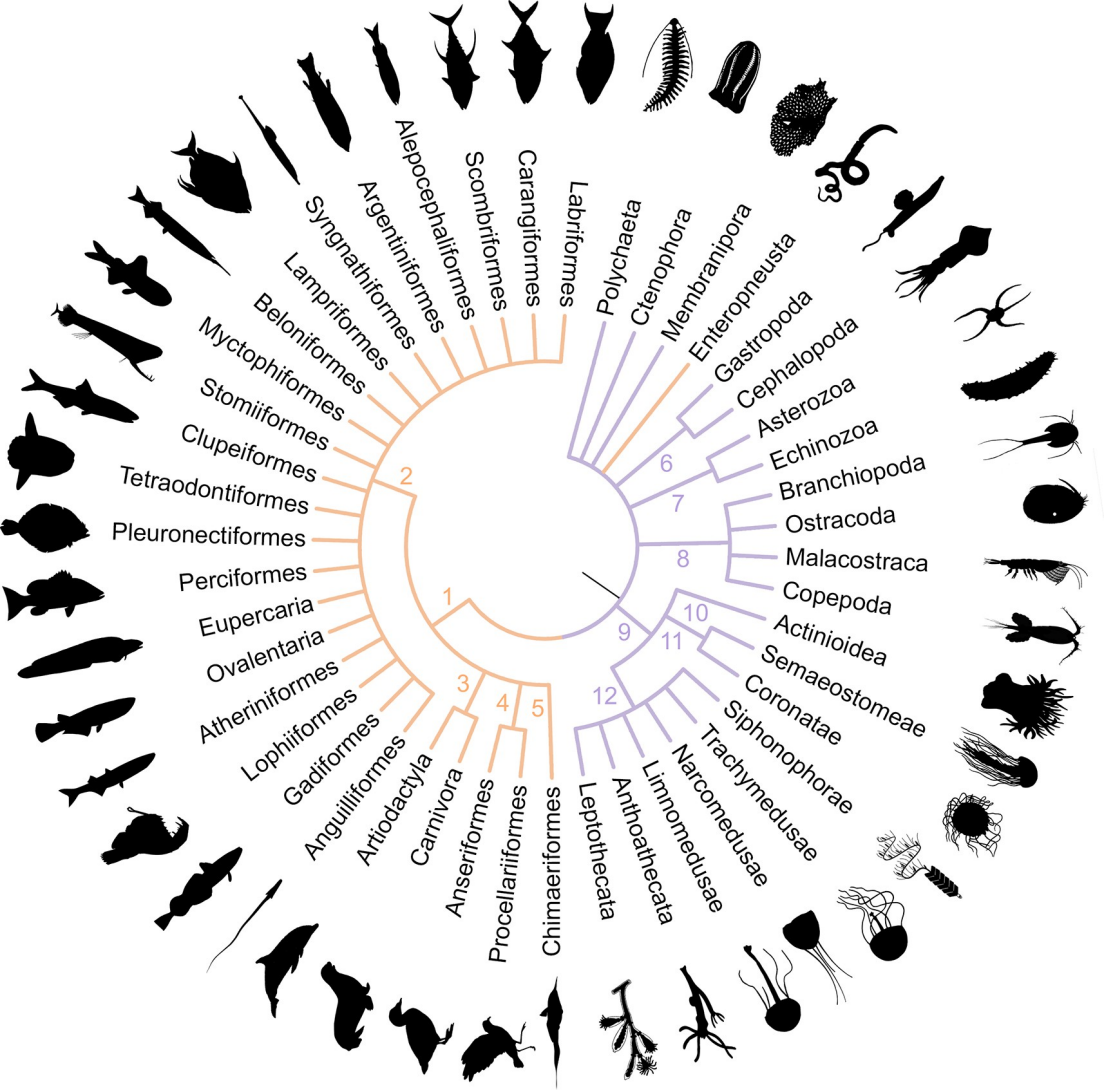
Simplified phylogenetic tree of taxonomic groups detected by eDNA methods. Groups identified using 12S are indicated in orange (Vertebrates: Phylum Chordata (1); Classes Actinopterygii (2), Mammalia (3), Aves (4), and Chondrichthyes (5); Hemichordates: Phylum Enteropneusta) and groups identified using COI are indicated in purple (Invertebrates: Phyla Ctenophora, Mollusca (6), Echinodermata (7), Cnidaria (9); Subphylum Crustacea (8); Classes Anthozoa (10), Scyphozoa (11), Hydrozoa (12), Polychaeta; Genus Membranipora). Branch lengths are not to scale.

In the 12S dataset, 81 “unique taxa” (unique ASV identifications at any taxonomic level) were identified from 26 taxonomic orders including 58 taxa identified to the species level (Fig 2 and S1 Table in S1 File). Of the taxa identified to the species level, there were 48 fishes, eight mammals, one hemichordate, and one bird. Clupeiformes accounted for the most reads (28% of total), as well as 15% of ASVs and 6% of unique taxa. Myctophiformes accounted for 23% of reads, 21% of ASVs, and had the most unique taxa of any group (16% of total). The 12S dataset identified fewer unique taxa than the COI dataset but generally resolved diversity at a finer taxonomic level with reads spread more evenly across taxonomic groups (S1 Table in S1 File). In the COI dataset, 126 unique taxa were identified including 35 taxonomic orders represented by 102 taxa identified to the species level (Fig 2, S1 Table in S1 File). Among the taxa identified to species, there were 35 Cnidarians, 29 Crustaceans, and 28 Polychaetes. Copepoda was the most dominant group, accounting for 65% of reads, 58% of ASVs, and 20% of unique taxa. Polychaeta had the most unique taxa of any group, accounting for only 2% of reads and 5% of ASVs, but 26% of unique taxa.

A total of 27 MOCNESS net samples were included in the methods comparison (Table 1). Sixty-eight species from 17 taxonomic groups of vertebrates and 153 unique taxa from 26 taxonomic groups of invertebrates represented by 49 species were detected in MOCNESS samples (Table 2). Stomiiformes, Myctophiformes, and Osmeriformes were the dominant vertebrate orders, representing 21%, 18%, and 17% of MOCNESS presences, respectively. Malacostraca, Siphonophorae, and Trachymedusae were the dominant invertebrate groups, representing 11%, 10%, and 9% of sample presences, respectively.

The ability to assign taxonomy to ASVs and compare taxonomy to MOCNESS data was limited by genetic reference libraries for both primer datasets. When matched to the MIDORI2 (12S, GenBank 252 updated October 2022) and MetaZooGene (COI, GenBank 254 updated February 2023) reference databases, 55% and 86% of ASVs had a hit to the species level for 12S and COI, respectively. Of the unique taxa identified from MOCNESS, 25% of vertebrate and 16% of invertebrate taxa were absent from MIDORI2 and MetaZooGene databases, respectively.

### eDNA and MOCNESS methods sample distinct assemblages

**Fig 3:**
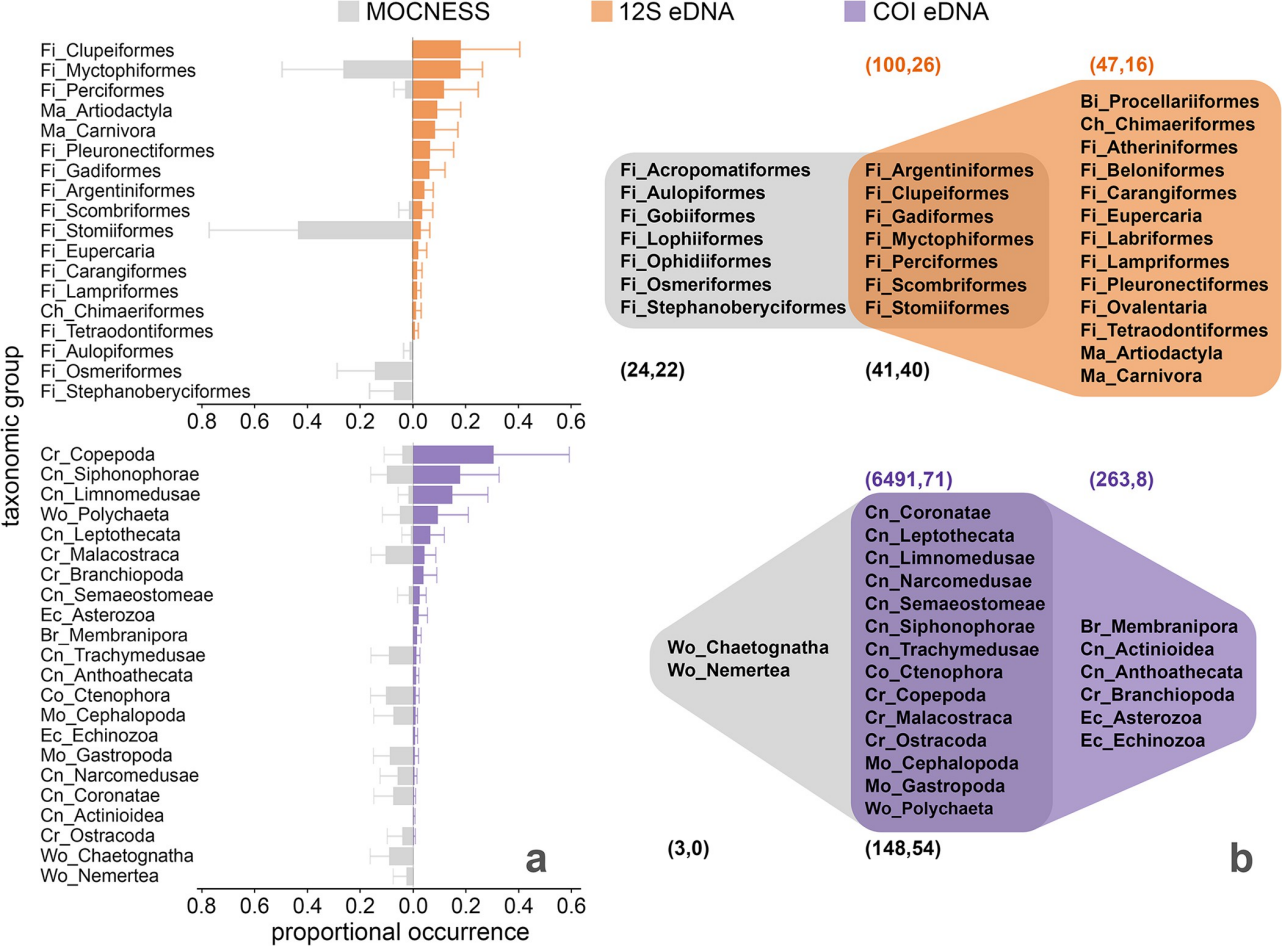
eDNA sampled a higher diversity of taxonomic groups than MOCNESS. **(a)** Mean proportional occurrence (± SD) of taxonomic groups in co-located MOCNESS (n = 27 nets) and eDNA samples from 0–1,750 m for vertebrate (n = 211 12S samples) and invertebrate groups (n = 244 COI samples). Bar charts include taxonomic orders with mean proportional occurrences greater than 0.01. **(b)** Venn diagrams show a higher richness of taxonomic groups sampled by eDNA than MOCNESS for both primers, but a higher number of vertebrate species were detected by MOCNESS. Number pairs describe total and species-level taxonomic richness for each method (number of ASVs for eDNA and unique taxonomic assignments for MOCNESS, number of species). Taxonomic group type key: Fi = bony fish, Ch = cartilaginous fish, Ma = mammal, Bi = bird; Cn = cnidarian, Cr = crustacean, Mo = mollusc, Br = bryozoan, Wo = worm, Ec = echinoderm.

Animal communities defined by proportional occurrence of taxonomic groups detected by eDNA and net-based sampling via MOCNESS were not significantly correlated across zones and depth groups (Mantel, *r* = 0.026, *p* = 0.393). Variability in proportional occurrence for all taxonomic groups within each method was similar across zones and depths (S2 Fig in S1 File) and were averaged across all samples according to each method (Fig 3A). The two methods shared representation of seven vertebrate (25% of 12S groups) and 14 invertebrate taxonomic groups (64% of COI groups, Fig 3B).

eDNA methods detected a greater richness of taxonomic groups than MOCNESS (Fig 3B), with 19 taxonomic groups exclusively detected in eDNA samples, versus nine groups exclusively observed in MOCNESS samples. Proportional occurrences of Clupeiformes, marine mammals (Artiodactyla, Carnivora), Copepoda, Limnomedusae, Leptothecata, and Branchiopoda were higher in the eDNA dataset. Conversely, Stomiiformes, Osmeriformes, Stephanoberyciformes, Malacostraca, Trachymedusae, Ctenophora, Gastropoda, and Chaetognatha had higher proportional occurrences in the MOCNESS dataset (Fig 3A).

### ASV diversity varies by primer and taxonomy

**Fig 4:**
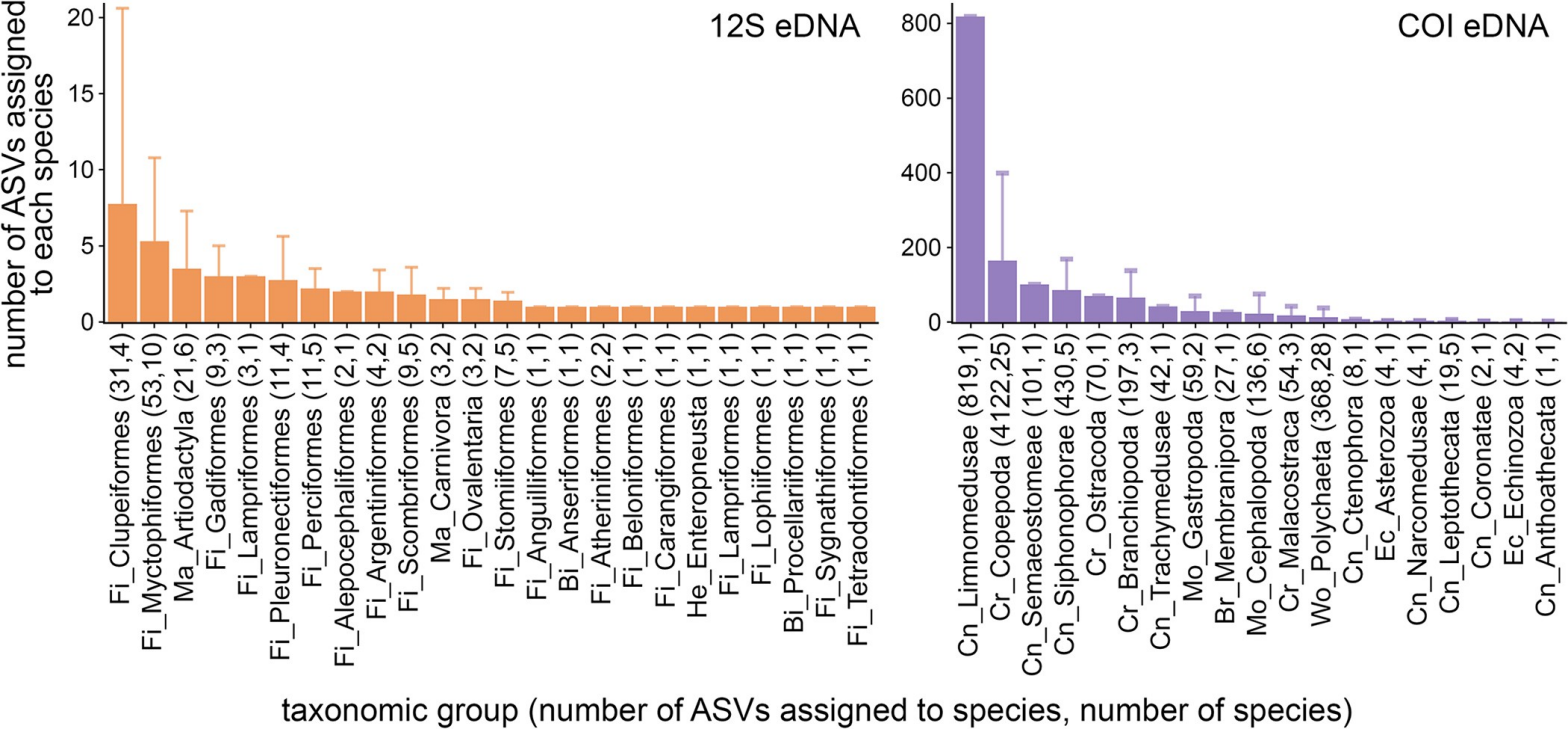
Species were often represented by multiple ASVs, with large variations across taxonomic groups. Bar plots display the mean number of ASVs per species within each taxonomic group, with error bars representing one standard deviation. Taxonomic group type key: Fi = bony fish, Ch = cartilaginous fish, Ma = mammal, Bi = bird, He = hemichordate, Cn = cnidarian, Cr = crustacean, Mo = mollusc, Br = bryozoan, Wo = worm, Ec = echinoderm.

Each invertebrate species was represented by an average of 78.1 ± 184.6 ASVs, with large variability both within and among taxonomic groups. Within the COI dataset, a single species of Limnomedusae (*Liriope tetraphylla*) was represented by 819 ASVs and the 25 species of copepods identified were represented by an average of 177 ± 244.9 ASVs (Fig 4). Species in four other animal groups (Semaetostomeae, Siphonophorae, Ostracoda, and Branchiopoda) were each represented by more than 50 ASVs on average. Each vertebrate species was represented by an average of 2.1 ± 1.7 ASVs. Species of Clupeiformes and Myctophiformes were each represented by more than five ASVs on average (Fig 4).

### eDNA concentrations and library completeness vary with depth

**Fig 5:**
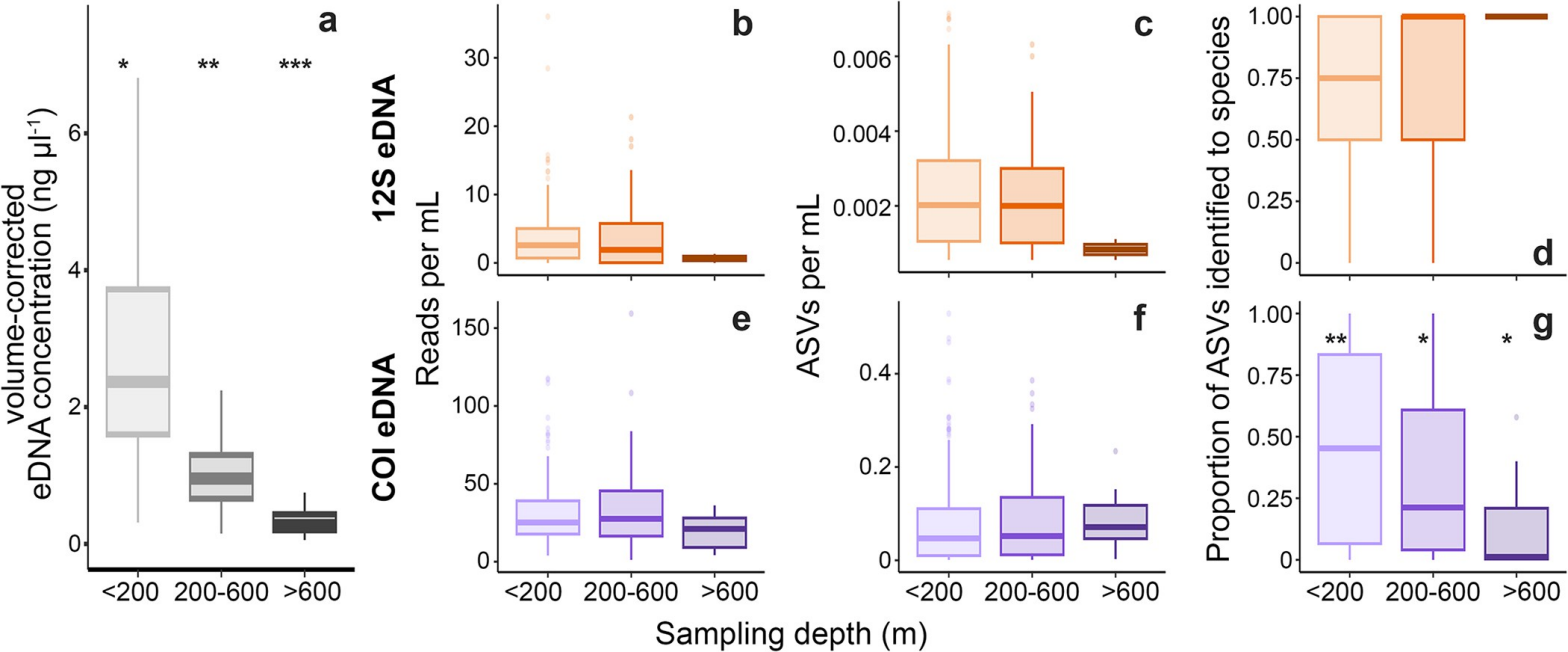
eDNA sample characteristics with respect to primer and sampling depth. **(a)** The concentration of DNA per sample decreased with depth **(**n = 433**)**. For samples with more than 1,000 reads, the number of reads per mL **(b,e)** and total ASVs per mL of seawater **(c,f)** were not meaningfully variable across depth bins. The proportion of ASVs assigned to species **(d,g)** decreased with depth for COI samples (n = 331), with 12S samples (n = 231) having higher proportions of species-level identifications than COI across depths. Statistical differences among depth bins (Games-Howell, *p* <0.05) are indicated by the number of asterisks. Results of global and *post hoc* tests describing the effects of depth bin on each eDNA metric are given in Table 3.

eDNA concentrations decreased with depth (Welch’s ANOVA, F = 590.65, *p* < 2.2 x 10^−16^, Table 3) and were an order of magnitude higher on average in samples collected shallower than 200 m (3.60 ng μl^-1^) than in samples collected deeper than 600 m (0.32 ng μl^-1^, Fig 5A and S3 Fig in S1 File). The numbers of reads and ASVs per mL were consistently higher in COI than 12S samples but there was no clear variability in the numbers of reads or ASVs per mL across sampling depth bins for either 12S or COI samples (Kruskal-Wallis, *X*^*2*^ = 0.83–5.89, *p* = 0.05–0.66, Table 3, Fig 5). After read- and sampling-time thresholding, only two 12S samples were collected deeper than 600 m and sampling depth was not a clear predictor of the proportion of ASVs identified to species in the 12S samples (Fig 5C). Fewer than 50% of all COI ASVs per sample could be identified to species (Fig 5F). COI samples collected shallower than 200 m had a higher proportion of ASVs identified to species than samples collected deeper than 200 m (Games-Howell, t = 2.80–3.17, *p* = 0.02–0.04).

**Table 3:** Summary of the effect of sampling depth on eDNA metrics.

	*Test*	*statistic*	*p-value*
***All samples (n = 433)***			
*DNA concentration (ng μl* ^*-1*^ *)*	Welch’s ANOVA	*F* = 590.58	**< 2.2 x 10** ^**−16**^
	GH <200:200–600	*t* = 17.09	**7.67 x 10** ^**−13**^
	GH <200:>600	*t* = 34.46	**2.11 x 10** ^**−10**^
	GH 200–600:>600	*t* = 10.91	**7.18 x 10** ^**−14**^
***12S (n = 231)***			
*Sequence reads per mL*	Kruskal-Wallis	*X*^*2*^ *=* 4.39	0.11
*ASVs per mL*	Kruskal-Wallis	*X*^*2*^ *=* 5.89	0.05
*Proportion ASVs to species*	Kruskal-Wallis	*X*^*2*^ *=* 2.44	0.29
***COI (n = 331)***			
*Sequence reads per mL*	Kruskal-Wallis	*X*^*2*^ *=* 2.72	0.26
*ASVs per mL*	Kruskal-Wallis	*X*^*2*^ *=* 0.83	0.66
*Proportion ASVs to species*	Welch’s ANOVA	*F* = 16.36	**4.58 x 10** ^**−3**^
	GH <200:200–600	*t* = 2.80	**0.02**
	GH <200:>600	*t* = 3.17	**0.04**
	GH 200–600:>600	*t* = 2.14	0.15

For most comparisons, a Kruskal-Wallis test was performed and Chi-squared values (*X*^*2*^) are reported. When data were non-normal and heteroscedastic among comparison groups, a Welch’s ANOVA and Games-Howell *post hoc* tests (GH) were performed. p-values < 0.05 are shown in bold text.

### Resolving large-scale spatial variability in animal assemblages detected with eDNA

**Fig 6:**
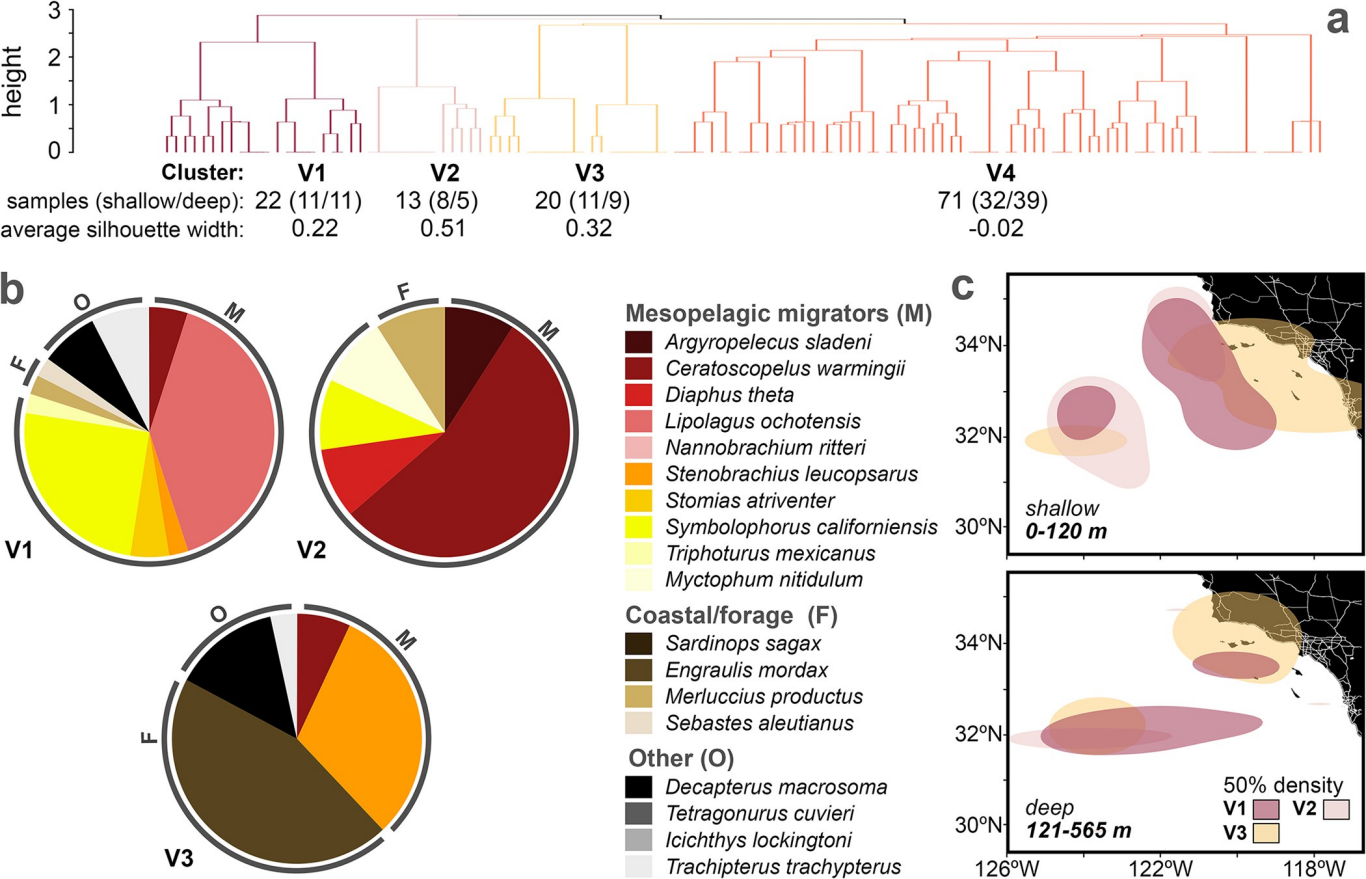
Hierarchical clustering of 12S samples reflects three spatially variable fish assemblages dominated by either coastal pelagic or mesopelagic migrator species. **(a)** Clustering was performed on 126 12S samples collected at night and only included species present in at least three samples. **(b)** Up to 10 of the most frequently occurring species are displayed for each cohesive cluster (average silhouette width >0.1). **(c)** Fifty percent probability density contours (“50% density”) demonstrate that nearshore clusters had the highest occurrences coastal forage species, and intermediate to offshore clusters were generally dominated by mesopelagic migrator species for both deep and shallow samples. Silhouette plots and additional probability density contour maps are given for each cluster in S5 and S6 Figs in S1 File, respectively.

Hierarchical clustering demonstrated that low volume eDNA samples have variable ability to resolve geographically distinct animal assemblages across primers and taxa. Of the samples included in our analyses, night-collected samples comprised 62% and 59% of the 12S and COI datasets, while day-collected samples comprised 17% and 14%, respectively. The remainder were collected during transitional periods between day and night. Using the elbow method, we identified four clusters in both eDNA datasets (S4 Fig in S1 File). In the 12S dataset, nighttime samples formed three cohesive clusters (V1-V3, mean silhouette widths 0.22–0.51) with 8.00 ± 4.36 species each (Fig 6A). Cluster V4 was not cohesive, with 65% of samples having negative silhouette widths (S5 Fig in S1 File). Each cohesive cluster was comprised of distinct assemblages of fishes with different biology and habitat preferences. The distributions of cohesive cluster samples largely aligned with known nearshore distributions of coastal forage species and offshore habitats of mesopelagic migrator taxa. Cluster V1 was dominated by the mesopelagic migrator species *Lipolagus ochotensis* (Bathylagidae) and *Symbolophorus californiensis* (Myctophidae) and contained shallow and deep samples. V2 was distributed mostly offshore in the southern part of our study area (S6 Fig in S1 File) and was dominated by an offshore myctophid species, *Ceratoscopelus warmingii* [61,62]. Cluster V3 was mostly contained shallow nearshore samples (Fig 6C and S6 Fig in S1 File) and was represented by the highest proportion of coastal forage species, dominated by the California anchovy, *Engraulis mordax*, and a myctophid with a relatively near-shore distribution in cooler habitats, *Stenobrachius leucopsaurus* ([63,64]; Fig 6B).

**Fig 7:**
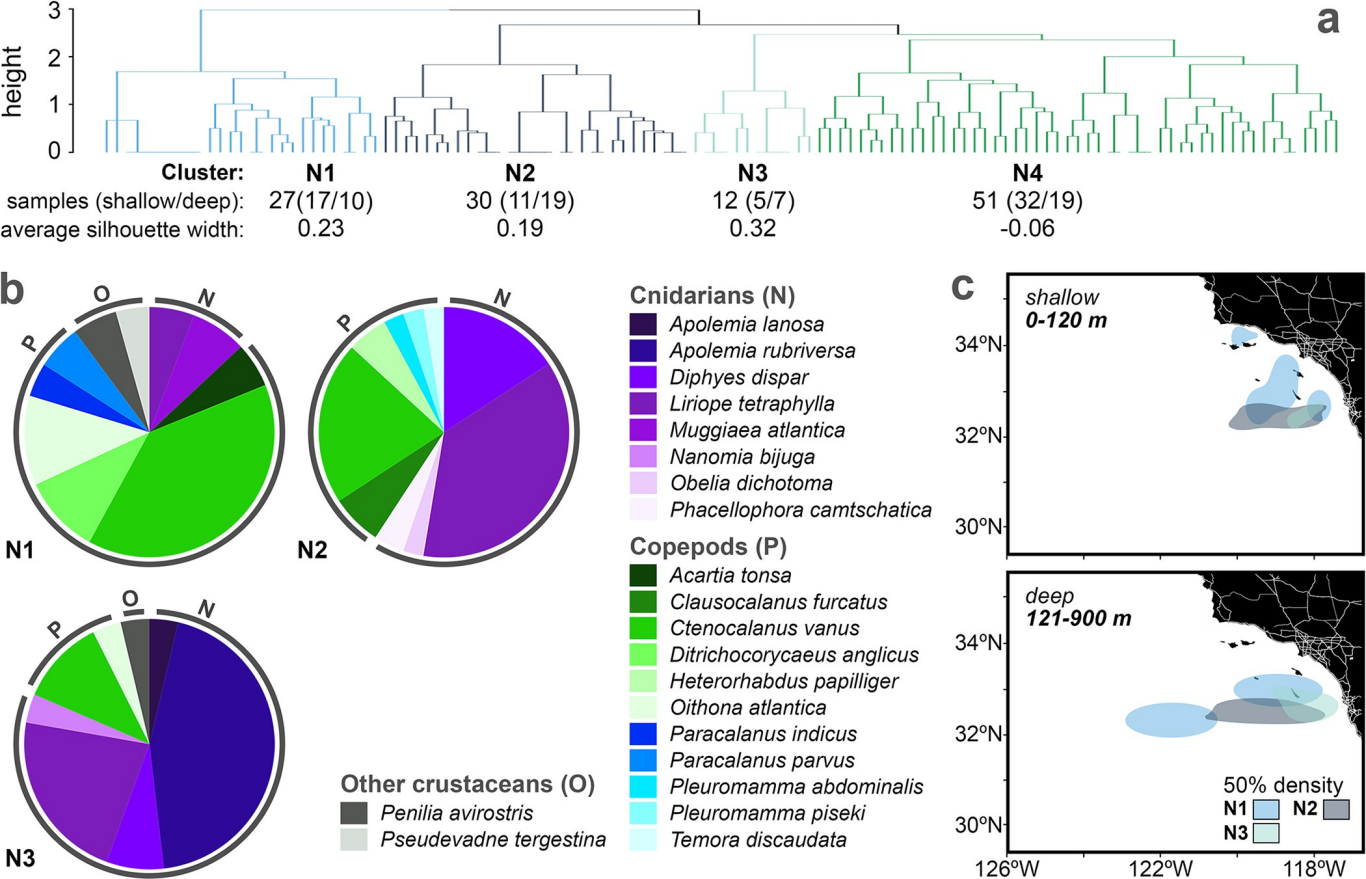
Hierarchical clustering of COI samples reflects three spatially variable invertebrate assemblages dominated by copepod and cnidarian species. **(a)** Clustering was performed on 120 COI samples collected at night and only included species present in at least three samples. **(b)** Up to 10 of the most frequently occurring species are displayed for each cohesive cluster (average silhouette width >0.1). **(c)** Fifty percent probability density contours (“50% density”) depict horizontal and vertical variability in the distribution of invertebrate assemblages. Silhouette plots and additional probability density contour maps are given for each cluster in S5 and S6 Figs in S1 File, respectively.

Hierarchical clustering also produced three cohesive clusters in the COI dataset (N1-N3, mean silhouette width 0.19–0.32) with 14.33 ± 9.07 species each (Fig 7A). Cluster N4 was not cohesive, with 71% of samples having negative silhouette widths (S5 Fig in S1 File). All cohesive clusters were primarily represented by copepods and cnidarians known to be abundant in the CCE [65]. Clusters N1 and N2 were the most diverse (Fig 7B and S7 Fig in S1 File). N1 was mostly comprised of an assemblage of copepods with peak occurrences in the upper 100 m of the water column in coastal and offshore waters off southern California (e.g., *Ctenocalanus vanus*, *Ditrichocorycaeus anglicus* [66,67]). N2 contained roughly equal frequencies of cnidarians and copepods, dominated by species distributed throughout the water column in the southern region of our study area (e.g., *Liriope tetraphylla* and *Ctenocalanus vanus* [68]). Cluster N3 was dominated by deep-sea physonect siphonophores, including *Apolemia rubriversa* and *Apolemia lanosa* [69,70].

Few species were represented per sample across clusters for both 12S (1.68 ± 0.28) and COI samples (2.70 ±0.40, S7 Fig in S1 File), and the number of species represented in each COI sample was substantially lower than the number of ASVs (35.30 ± 8.35, S2 Table in S1 File). Clusters V3 and N3 had the lowest overall species richness and highest mean frequency of species occurrence across samples (S7 Fig in S1 File). Clusters V4 and N4 had the highest richness relative to the other clusters (S7 Fig in S1 File) but had low cohesiveness among samples (S5 Fig in S1 File). Although each sample detected few species, combining many low diversity samples enabled the identification of geographically distinct assemblages.

## Discussion

We found that low volume eDNA samples can be broadly useful for assessing the diversity and distribution of pelagic animals. By combining sequence data from numerous low volume samples collected from a broad spatial area and spanning extensive depth horizons, we observed animal assemblages comprised of zooplankton, micronekton, and coastal forage fishes. These assemblages were consistently distinct from those detected with nets but reflected known biogeographic patterns for dominant taxa. In general, eDNA samples represented a lower diversity of vertebrate species and a higher diversity of invertebrate species than animals collected by the large MOCNESS, which could be explained by method-specific biases. We discuss how eDNA methods represent assemblages across habitat depths and propose future work that could improve the ability of low volume eDNA samples to describe pelagic zooplankton and micronekton diversity.

### Method-specific biases explain variability in assemblages sampled by eDNA and MOCNESS

Differences in the detection of animals via eDNA and MOCNESS methods were consistent among sampling zones and depths and were most notably driven by variability in the degree of size selectivity between methods. Since eDNA is detected at the molecular-, rather than the individual scale, it does not bias against size in the same way that net-based sampling does [71]. We detected mammals as well as small copepods and likely larvae or gametes via eDNA sampling: specimens which are generally too large or small, respectively, to be retained by 10 m^2^ MOCNESS sampling. Within the size ranges of zooplankton and micronekton, we also detected organisms using eDNA that are fragile and generally poorly sampled by nets (e.g., Cnidarians [4]) or mobile enough to avoid sampling (e.g., coastal forage fishes including Clupeiformes [72]). Although eDNA methods can sample taxa across a large size range, larger individuals are likely to shed more eDNA than smaller individuals of the same species, complicating the relationship between eDNA concentration and organism abundance [73].

Across taxonomic groups, species that were readily detected with eDNA methods are known to be abundant in the southern CCE (e.g., fishes: *Engraulis mordax*, *Nannobrachium ritteri*; dolphin: *Delphinus delphis*; copepod: *Ctenocalanus vanus*; medusa: *Liriope tetraphylla*). However, some abundant groups from the MOCNESS samples were notably rare in our eDNA datasets. Malacostracan crustaceans, particularly euphausiids and decapods, comprise a large proportion of pelagic biomass [2,4,74] can shed less DNA compared to fishes and gelatinous taxa [22] and represented a very small proportion of the COI ASVs and reads. Although we detected a very large number of cnidarian ASVs, most cnidarians groups were underrepresented in the COI eDNA dataset relative to their abundance in the MOCNESS dataset. This could stem from irregular shedding mechanisms among gelatinous organisms [22] or poor representation by the COI marker gene [75–78]. Ctenophores and molluscs have high rates of evolution in the mitochondrial COI gene, making it a less effective ‘universal’ barcode region for those groups [76,77,79]. For all taxa, positive identification of ASVs are directly dependent upon representation in verified reference libraries, which is known to be highly limited for ctenophores [80]. Moreover, incorrect taxonomy can be assigned to an ASV if it shares a barcode sequence with a closely related but distinct species.

All groups that were better represented by MOCNESS contained species that were not present in the genetic reference libraries. There is a clear need for improved verified reference libraries for abundant pelagic invertebrate groups (e.g., malacostracan crustaceans, cnidarians) to improve our ability to reliably assign species-level identifications to both specimens and sequences [81,82]. Recent efforts have greatly improved regional reference libraries for epipelagic and coastal fishes in the CCE [83], but there has been little focus on invertebrate groups relative to their high taxonomic diversity and abundance across the water column [2,4,74].

### Sampling depth affects species detection with eDNA

eDNA concentrations were an order of magnitude higher in shallow than in deep samples, consistent with observations from other pelagic systems [28,32]. Despite decreasing eDNA concentrations, we observed limited variability in the number of reads and ASVs per mL across the water column, suggesting that sampling depth did not affect our ability to detect diversity in the selected marker genes. There was no apparent variability in the proportion of 12S ASVs assigned to species across sampling depths. However, deeper-dwelling invertebrate taxa appear to be particularly underrepresented in COI reference libraries; the proportion of species-level ASV taxonomic assignments was three times higher in COI samples collected shallower than 200 m than in those collected deeper than 600 m.

Variability in the proportion of COI ASVs assigned to species across depths could also be driven by changes in community composition with depth. Although we were not able to assess variability in 12S library performance across our deepest samples, the rate at which fishes shed genetic material increases with metabolic rate [84,85], which generally decreases with depth of occurrence [86–88]. Some abundant deep pelagic fish groups, including members of the Stomiiformes, are scaleless, which may further reduce shedding rates and help explain their absence from the 12S dataset. Malacostracan crustaceans and cnidarians comprise increasing proportions of pelagic micronekton biomass with depth (e.g., [4,74]). Thus, poor representation of these groups in reference libraries greatly limits the realistic representation of deep pelagic animal assemblages. Continued efforts to generate barcode sequences for deep-pelagic invertebrates across multiple marker regions would drastically improve the utility of eDNA for broad detection of animal assemblages across habitat depths.

### Relating eDNA sequence diversity to assemblage diversity

By applying “universal” PCR primers for both vertebrates and invertebrates, we were able to detect diverse taxa across a large size range that typically requires extensive expertise and effort to morphologically identify with net-based sampling. Although these advantages make eDNA methods broadly useful for sampling animals, the lack of size selectivity and variability in suitability of genetic markers make it difficult to discern ecologically meaningful assemblages [89]. Among taxa that are well represented by the selected markers, there is clear variability in the relationship between ASV diversity and species diversity. We observed over two orders of magnitude variability in the number of COI ASVs per species across invertebrate groups, with 37 species represented by more than 50 ASVs (Fig 4). COI samples included in the clustering analyses contained thousands of reads and dozens of ASVs, but only represented ~2 species each (S2 Table and S7 Fig in S1 File). There were fewer ASVs per species among vertebrate groups detected in the 12S dataset. Community analyses that utilize ASVs instead of taxonomic IDs will overrepresent taxonomic diversity and could erroneously inflate variability among sampling sites and depths [90,91]. Clustering ASVs into operational taxonomic units (OTUs) based on percent identity thresholds is a common approach for mitigating variable resolution taxonomic assignments including multiple ASVs per species [28,92,93]. Blanco-Bercial et al. [93] showed high agreement between COI OTU diversity and species diversity in copepods, but the relationship is poorly studied across animal groups and marker regions and OTUs are not necessarily replicable across studies [37]. Conversely, species represented by extremely high numbers of ASVs (e.g., the medusa *Liriope tetraphylla*) may represent misidentifications that could underrepresent taxonomic diversity. Continued improvements of reference libraries (as in [83]) will enable better taxonomic assignments for poorly sampled groups and broader comparisons across studies using species-level eDNA analyses.

### Low volume eDNA samples can resolve some biogeographic structure in the pelagic

Our findings suggest that while low volume eDNA samples may not be appropriate for exhaustive diversity assessments, they can resolve large-scale spatial variability in the presences of abundant taxa. More than 50 vertebrate and invertebrate species were represented in our taxonomically restrictive clustering analyses and each cohesive cluster (n = 6) contained an average of eight species for 12S and 14 species for COI. Large numbers of small volume samples increased the species richness in our dataset, analogous to the increases seen with larger sample volumes and replicates in other studies [28,94,95]. Some geographical and taxonomic overlap was observed between most clusters, which could be explained by the minimal number of species within each sample (S7A and S7D Fig in S1 File) and variable spatial ranges of the taxa represented. Although cross-contamination among samples could also obscure spatial structure in our samples, clusters generally reflected the known distributions of abundant taxa detected with both primers. Samples collected near the coast in shallow waters were dominated by fishes, copepods, and cnidarians with known coastal distributions, while offshore and deep samples were generally comprised of vertically-migrating fishes and midwater cnidarians known to occur in offshore habitats. Low volume eDNA samples may currently be best suited for monitoring abundant species across taxonomic groups that are well represented by universal primers. If the relationships between these taxa, environmental conditions, and broader animal communities are known or can be quantified (e.g., [96,97]), taxa detected by low volume eDNA analyses could be treated as indicators of broader animal community variability [98,99].

### Towards animal assemblage monitoring with low volume eDNA samples

It is likely that monitoring of pelagic animals with eDNA will often rely on low volume water samples, either from existing sampling programs, cruises of opportunity, and even using dedicated sampling with autonomous underwater vehicles [100–102]. Thus, optimizing low volume eDNA methods is critical for supporting pelagic monitoring efforts. Given low agreement in species diversity between eDNA replicates [28,91,94], the retention of material for future replication is not necessary and use of entire eDNA filters would increase the quantity of genetic material and number of species detected per sample. Further, setting a higher target sequencing depth could also be a prudent way to increase the number of reads generated and species detected in low volume samples [103]. Application of an additional genetic marker to target malacostracan crustaceans, such as an 18S primer, has demonstrated improved capability of capturing this important component of the pelagic ecosystem [104], partially due to the larger number of reference sequences available for 18S compared to COI [105]. Implementation of a larger number of markers to better represent the diversity of pelagic animals (especially malacostracan crustaceans, cnidarians, and molluscs) would drastically improve taxonomic coverage and maximize the value of limited seawater samples. While large volume sampling and greater sequencing depths is clearly necessary for more comprehensive biodiversity inventories, continued improvement of reference libraries could combat issues of limited material in low volume samples. In the meantime, focusing on abundant “indicator” species may reveal broad responses to environmental variability [106,107] and allow for monitoring of pelagic animal assemblages with low volume eDNA sampling.

## Supporting information

S1 File All supplementary figures, tables, and methods. The supplementary material contains seven figures (S1-S7 Figs), two tables (S1 and S2 Tables), and methods information including descriptions of the primers used, PCR recipes, and PCR cycle conditions.
(PDF)

S1 Dataset All data required to replicate each of the analyses and figures presented in this study. This Excel data file consists of nine separate worksheets including a READ_ME that describes the data presented and the figures and tables that were generated from each corresponding sheet.
(XLSX)
