# Supplementary material for: Using low volume eDNA methods to sample pelagic marine animal assemblages
Source: PLoS One. 2024 May 15;19(5):e0303263. doi: 10.1371/journal.pone.0303263 (PMC11095688; doi:10.1371/journal.pone.0303263)
Supplement: S1 File — The supplementary material contains seven figures (S1-S7 Figs), two tables (S1 and S2 Tables), and methods information including descriptions of the primers used, PCR recipes, and PCR cycle conditions. (PDF) [file pone.0303263.s001.pdf]

Using low volume eDNA methods to sample pelagic marine animal assemblages

Michelle E. Dan[1], Elan J. Portner[1], Jeff S. Bowman[1], Brice X. Semmens[2], Sarah M. Owens[3], Stephanie M. Greenwald[3], and C. Anela Choy[1*]

[1] Integrative Oceanography Division, Scripps Institution of Oceanography, University of California San Diego, San Diego, California, United States of America

[2] Marine Biology Division, Scripps Institution of Oceanography, University of California San Diego, San Diego, California, United States of America

[3] Biosciences Division, Argonne National Laboratory, Lemont, Illinois, United States of America

* Corresponding author

Email: anela@ucsd.edu (CAC)

Supporting information

This document contains Supplementary Figures S1, S2, S3, S4, S5, S6, and S7; Supplementary Tables S1 and S2; Datafile S1 caption; and Supplementary Methods Information.

Figures

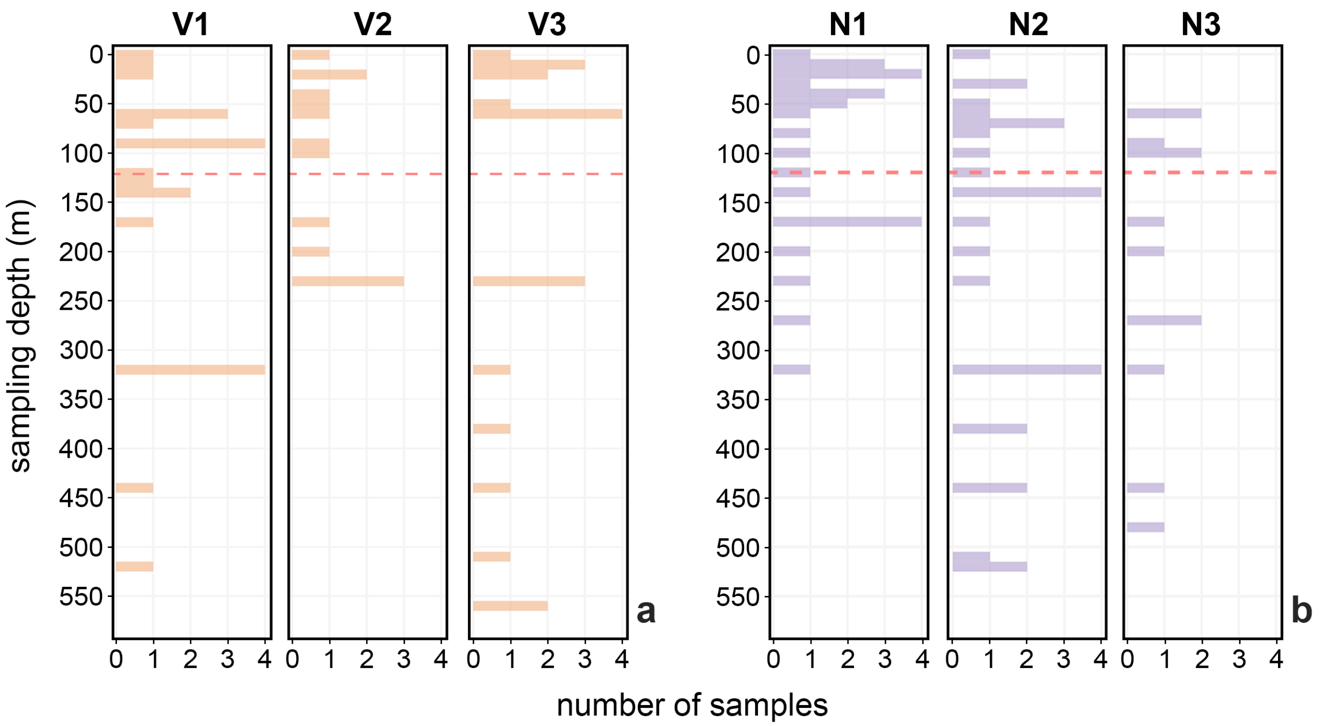

Fig S1. Sample depths for all cohesive clusters. Depth distributions of 12S (**a**) and COI (**b**) samples in each cohesive cluster. Red dashed line at 120 m represents distinction between "shallow" and "deep" samples used in the clustering analyses. Note that a single sample collected at 900 m is present in cluster N2 but was cropped out of the plot to improve visualization of sampling depths among clusters.

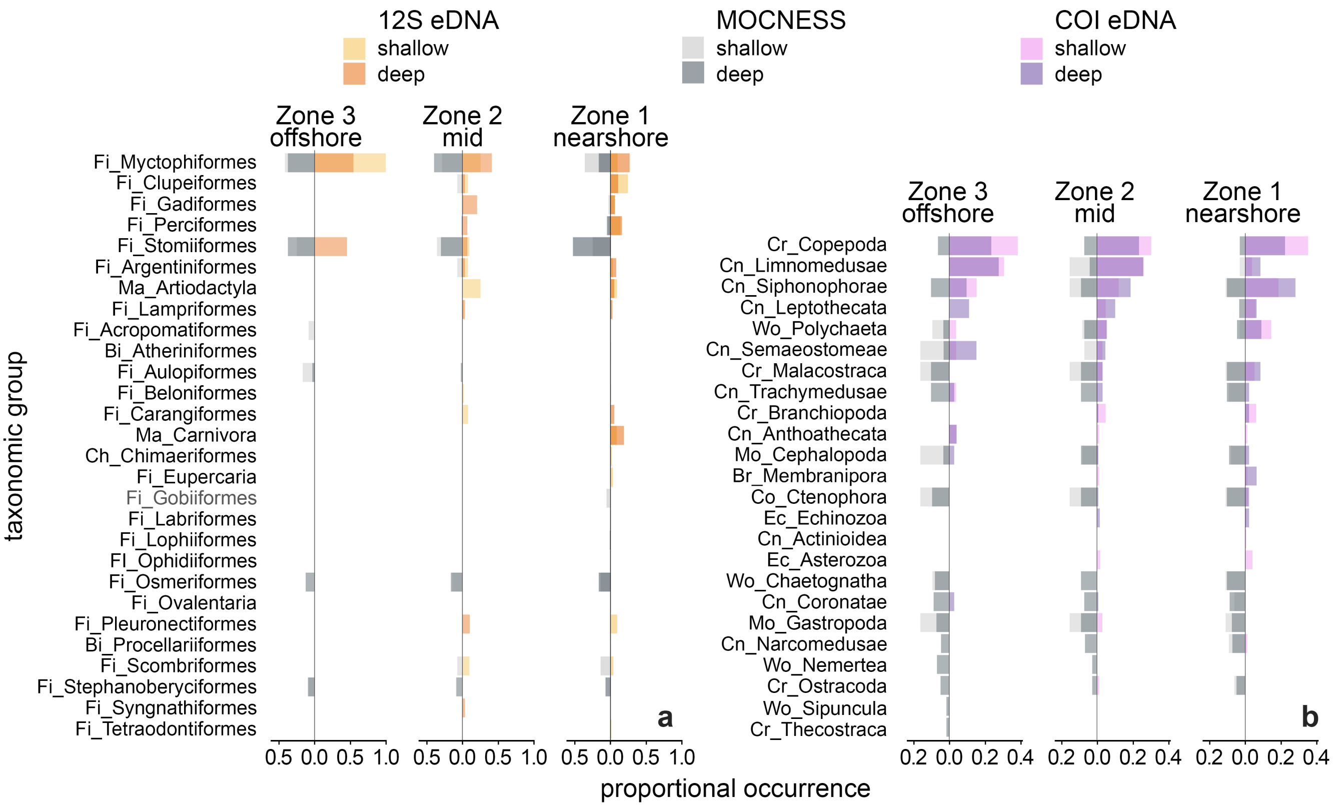

Fig S2. Comparison of assemblages sampled by MOCNESS and eDNA methods across zones and depths. 12S eDNA (**a**), COI eDNA (**b**) and MOCNESS methods observed distinct assemblage compositions across sampling zones and depths. Proportional occurrences of all taxonomic groups in co-located eDNA and MOCNESS samples are plotted across sampling zones (see Fig 1) and depth bins (shallow: 0-200 meters; deep: 201-1750 meters). The taxonomic group type key is as follows: Fi = bony fish, Ch = cartilaginous fish, Ma = mammal, Bi = bird; Cn = cnidarian, Cr = crustacean, Mo = mollusc, Br = bryozoan, Wo = worm, Ec = echinoderm.

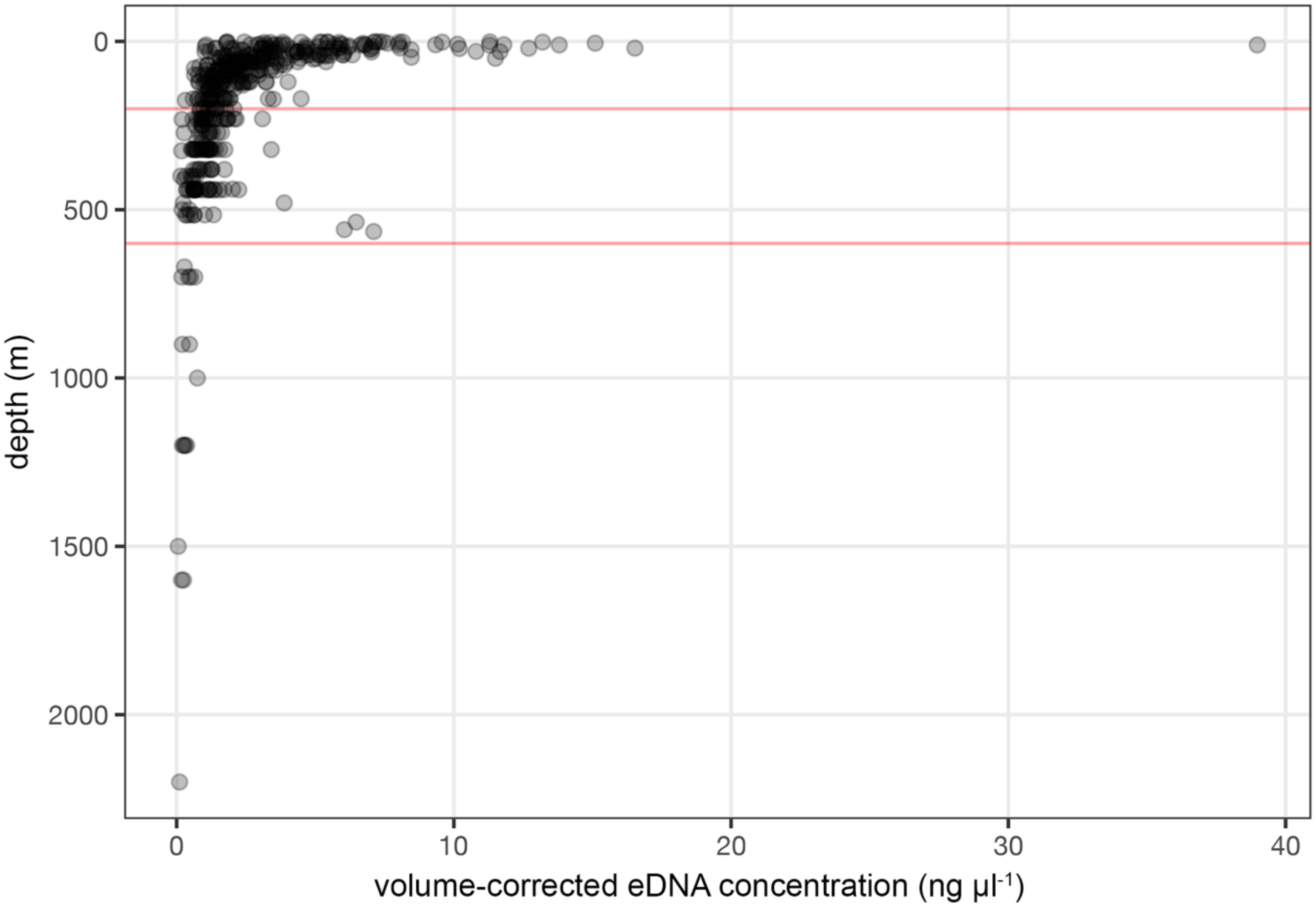

Fig S3. Volume-corrected eDNA concentration for all samples. Red lines indicate breaks at 200 m and 600 m between depth bins used to quantify variability in eDNA metrics with sampling depth (n = 433, see Fig 5, Table 3).

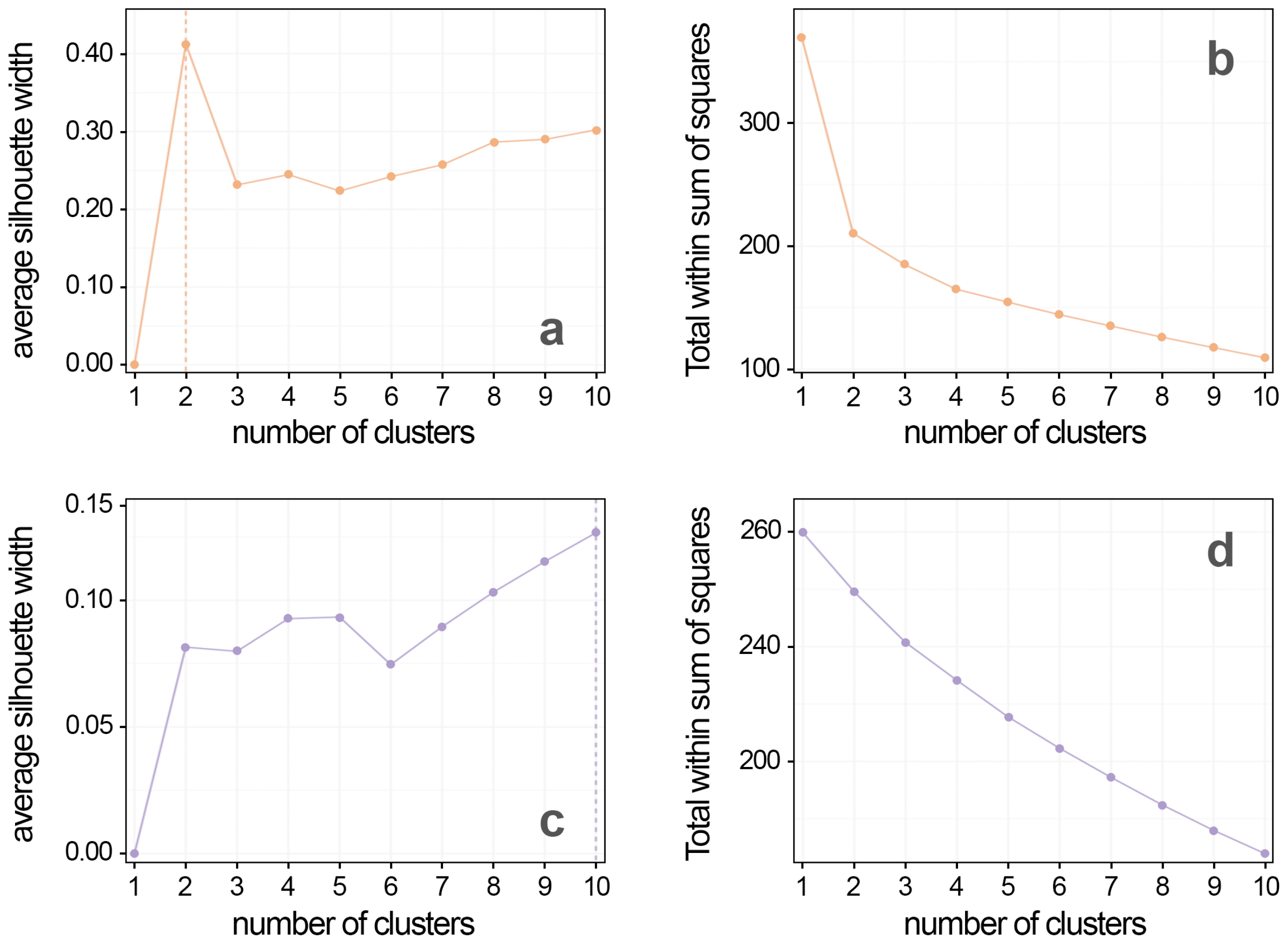

Fig S4. Graphical methods to support selection of an "optimal" number of clusters. Silhouette (**a, c**) and within group sum of squares (**b, d**) metrics for the 12S dataset (**a, b**) and COI dataset (**c, d**). Based on both methods of separation distance and the number of samples present in each cluster, we determined the optimal number of clusters was four for both the 12S and COI datasets to provide a parsimonious but informative level of sample partitioning.

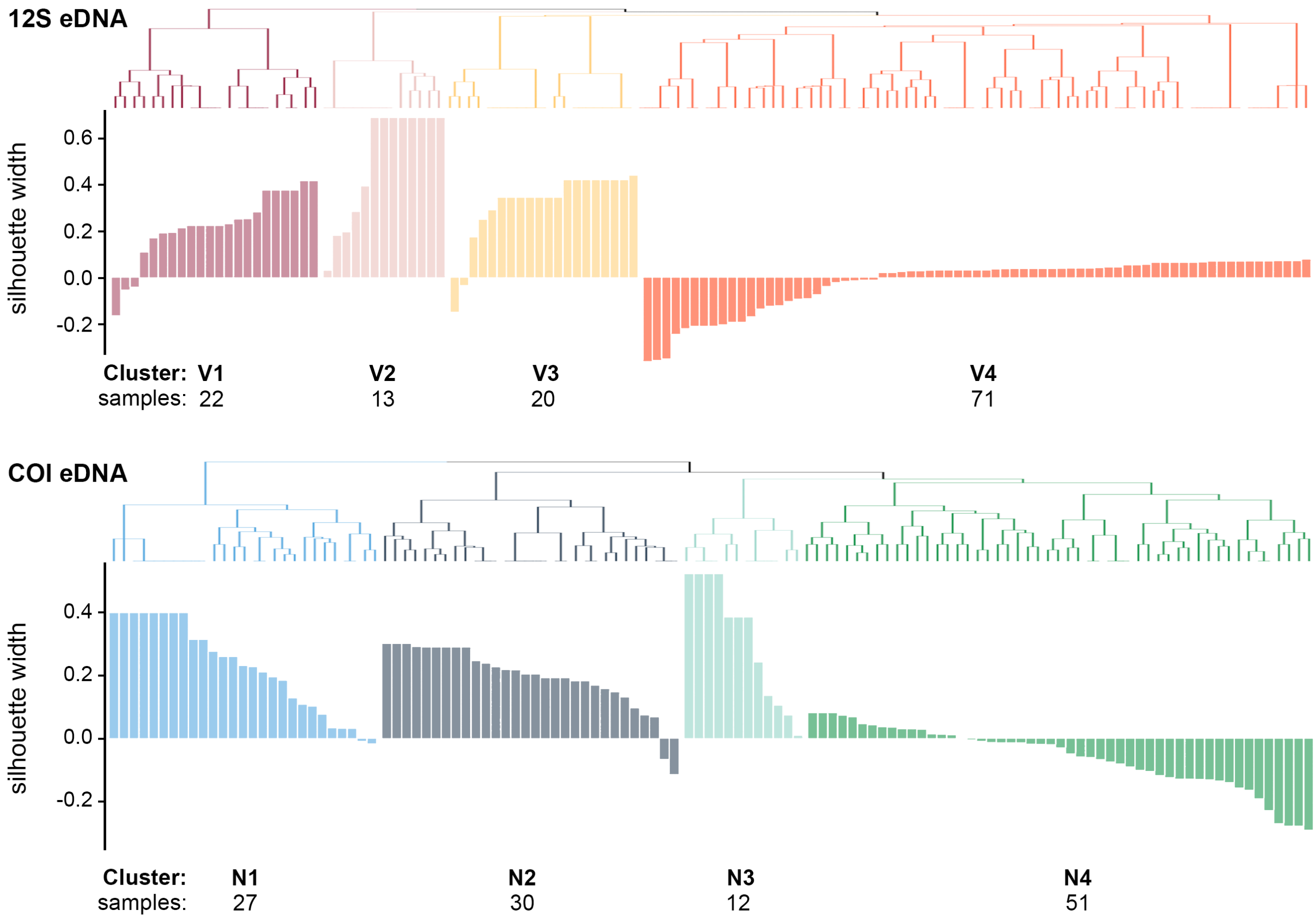

Fig S5. Dendrograms and silhouette widths of hierarchical clusters. Higher proportions of negative silhouette widths in Clusters V4 and N4 indicated low separation distances, or cohesiveness, between samples. Note that samples for both the 12S (top) and COI datasets (bottom) are presented in order of descending silhouette widths within each cluster and do not necessarily represent the corresponding sample indicated on the dendrogram.

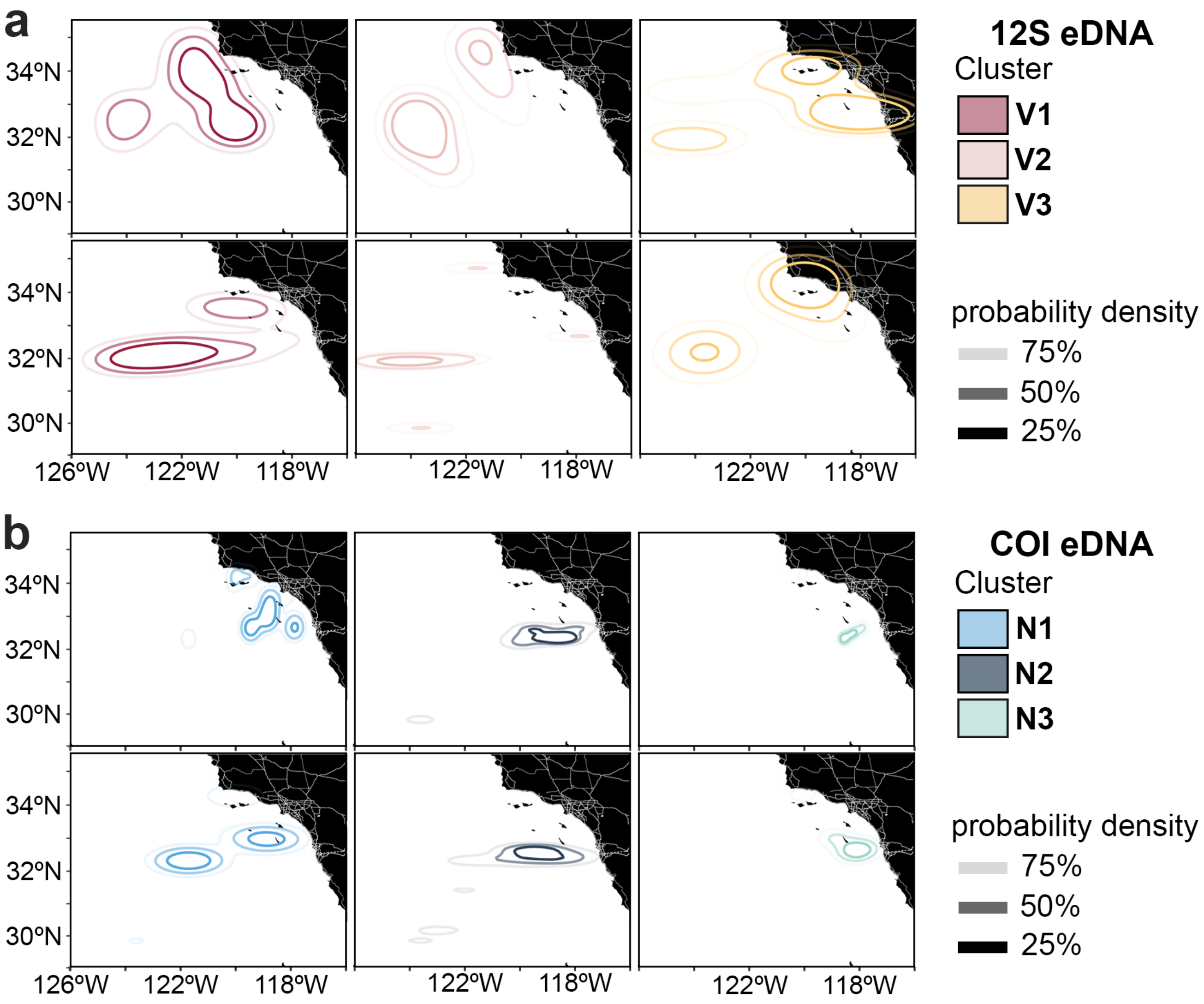

Fig S6. Probability density contour maps. The 25%, 50%, and 75% sample probability density contours are represented for each cohesive cluster in the 12S (**a**) and COI (**b**) datasets.

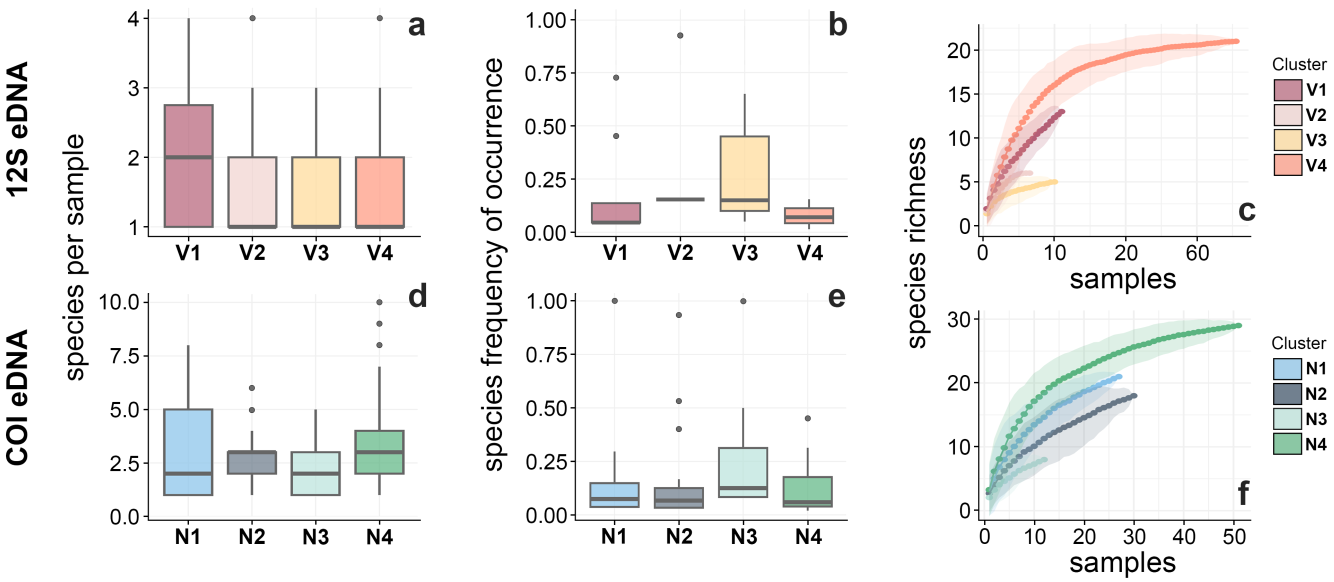

Fig S7. Characteristics of samples included in clustering analyses. The number of species per sample **(a, b)** was relatively low across clusters for both the 12S (top row) and COI (bottom row) datasets. Each cohesive cluster contained few species present in more than 50% of samples, with most species being present in fewer than 25% of samples per cluster **(b, e)**. Species accumulation curves **(c, f)** demonstrate that the non-cohesive clusters (V4 and N4) have higher species richness than more cohesive clusters (V1-V3, N1-N3).

Tables and Table Captions

Table S1. Levels of taxonomic assignment and numbers of representatives eDNA and MOCNESS datasets. eDNA "assignment level" values in "Species":"Phylum" columns refer to the most specific resolution of taxonomic assignment for an ASV (*i.e.*, an ASV assigned to the order level and *not* to the family level). eDNA "unique representatives" refer to the total number of distinct assignments at each level.

		**Total ASVs**		**Species**	**Genus**	**Family**	**Order**	**Class**	**Phylum**
**eDNA**	**12S**	245	assignment level	146	46	43	10	0	0
			unique representatives	58	59	42	26	6	2
	**COI**	7,971	assignment level	7,215	87	40	355	0	0
			unique representatives	102	93	14	35	8	8
		**Total unique taxa**		**Species**	**Genus**	**Family**	**Order**	**Class**	**Phylum**
**MOCNESS**	**vertebrate**	68	unique representatives	68	57	26	17	1	1
	**invertebrate**	153	unique representatives	49	88	67	26	13	9

Table S2: Summary of the average (± SD) numbers ASVs and reads per sample in each hierarchical cluster.

Primer	Cluster	Average ASV count (± SD)	Average reads (± SD)
12S	V1	2.05 (1.00)	3239.82 (4820.34)
12S	V2	1.77 (1.09)	1594.54 (1610.88)
12S	V3	1.70 (0.92)	2970.90 (5208.22)
12S	V4	1.82 (0.98)	3162.58 (3737.41)
COI	N1	36.22 (38.49)	2059.85 (2899.43)
COI	N2	34.90 (51.25)	1244.80 (2677.38)
COI	N3	24.83 (31.89)	910.92 (1246.52)
COI	N4	45.24 (72.82)	1441.57 (2257.39)
COI	N5	19.81 (26.84)	1256.19 (1644.61)

Supplementary Datafile Caption

Dataset S1. **All data required to replicate each of the analyses and figures presented in this study.** This Excel data file consists of nine separate worksheets including a READ_ME that describes the data presented and the figures and tables that were generated from each corresponding sheet.

Supplementary Methods Information

Primer Sequences. We applied *ecoPrimers* amplifying a 73-110 bp fragment of the 12S-V5 gene (5′ ACTGGGATTAGATACCCC-3' and 5′ TAGAACAGGCTCCTCTAG -3') [1] and the mlCOIintF and jgHCO2198 primers amplifying a 313 bp fragment of the metazoan COI gene (5'-GGWACWGGWTGAACWGTWTAYCCYCC-3' and 5'-TAIACYTCIGGRTGICCRAARAAYCA-3') [2].

PCR Reaction Recipes. The reaction conditions for the 12S primer set are as follows: PCR was run in a 25-μL reaction containing 5.5 μL DNA extract, 12.5 μL AccuStart master mix (Quantabio, Beverly, MA), 1 μL of each the forward and reverse primer (10 μM each), and 5 μL molecular-biology-grade water. The reaction conditions for the COI primer set are as follows: PCR was run in a 20-μL reaction containing 2 μL DNA extract, 14 μL AccuStart master mix, 1 μL of each of the forward and reverse primer (10 μM each), and 5.5 μL molecular-biology-grade water.

PCR Cycle Conditions. The PCR cycling conditions for the 12S-V5 primer set was as follows: 95°C for 5 min followed by 40 cycles of 95°C for 15 s, 55°C for 30 s and 72°C for 30 s. The PCR cycling conditions for the COI PCR set was as follows: 95ºC for 1 min, 16 cycles of 95ºC for 10 s, 62ºC for 30 s, and 72ºC for 1 min, followed by 20 cycles of 95ºC for 10 s, 46ºC for 30 S, 72ºC for 1 min, then 72ºC for 7 min.

References

1. Riaz T, Shehzad W, Viari A, Pompanon F, Taberlet P, Coissac E. ecoPrimers: inference of new DNA barcode markers from whole genome sequence analysis. Nucleic Acids Res. 2011;39: e145. doi:10.1093/nar/gkr732

2. Leray M, Yang JY, Meyer CP, Mills SC, Agudelo N, Ranwez V, et al. A new versatile primer set targeting a short fragment of the mitochondrial COI region for metabarcoding metazoan diversity: application for characterizing coral reef fish gut contents. Front Zool. 2013;10: 34. doi:10.1186/1742-9994-10-34
